# Interferon-gamma polymorphisms and risk of iron deficiency and anaemia in Gambian children

**DOI:** 10.12688/wellcomeopenres.15750.2

**Published:** 2020-06-02

**Authors:** Kelvin M. Abuga, Kirk A. Rockett, John Muthii Muriuki, Oliver Koch, Manfred Nairz, Giorgio Sirugo, Philip Bejon, Dominic P. Kwiatkowski, Andrew M. Prentice, Sarah H. Atkinson

**Affiliations:** 1 Kenya Medical Research Institute (KEMRI) Centre for Geographic Medicine Coast, KEMRI-Wellcome Trust Research Programme, Kilifi, Kenya; 2 Wellcome Centre for Human Genetics, Nuffield Department of Medicine, University of Oxford, Oxford, UK; 3 Open University, KEMRI-Wellcome Trust Research Programme – Accredited Research Centre, Kilifi, Kenya; 4 Infection Medicine, The University of Edinburgh, Edinburgh, UK; 5 Department of Internal Medicine II, Medical University Innsbruck, Innsbruck, Austria; 6 Perelman School of Medicine, University of Pennsylvania, Philadelphia, USA; 7 Centre for Tropical Medicine and Global Health, Nuffield Department of Medicine, University of Oxford, Oxford, UK; 8 Wellcome Sanger Institute, Hinxton, Cambridge, UK; 9 Big Data Institute, Li Ka Shing Centre for Health Information and Discovery, University of Oxford, Oxford, UK; 10 Medical Research Council Unit The Gambia at the London School of Hygiene and Tropical Medicine, Banjul, The Gambia; 11 Department of Paediatrics, University of Oxford, Oxford, UK

**Keywords:** Interferon-gamma, malaria, iron deficiency, anaemia, ferritin, hepcidin, zinc protoporphyrin, transferrin saturation, iron, IFNG, genetic polymorphisms, Africa, children

## Abstract

**Background**: Anaemia is a major public health concern especially in African children living in malaria-endemic regions. Interferon-gamma (IFN-γ) is elevated during malaria infection and is thought to influence erythropoiesis and iron status. Genetic variants in the IFN-γ gene *(IFNG*) are associated with increased IFN-γ production. We investigated putative functional single nucleotide polymorphisms (SNPs) and haplotypes of *IFNG* in relation to nutritional iron status and anaemia in Gambian children over a malaria season.

**Methods: **We used previously available data from Gambian family trios to determine informative SNPs and then used the Agena Bioscience MassArray platform to type five SNPs from the *IFNG* gene in a cohort of 780 Gambian children aged 2-6 years. We also measured haemoglobin and biomarkers of iron status and inflammation at the start and end of a malaria season.

**Results: **We identified five *IFNG* haplotype-tagging SNPs ( *IFNG*-1616 [rs2069705], *IFNG*+874 [rs2430561], *IFNG*+2200 [rs1861493], *IFNG*+3234 [rs2069718] and *IFNG*+5612 [rs2069728]). The *IFNG*+2200C [rs1861493] allele was associated with reduced haemoglobin concentrations (adjusted β -0.44 [95% CI -0.75, -0.12]; Bonferroni adjusted P = 0.03) and a trend towards iron deficiency compared to wild-type at the end of the malaria season in multivariable models adjusted for potential confounders. A haplotype uniquely identified by *IFNG*+2200C was similarly associated with reduced haemoglobin levels and trends towards iron deficiency, anaemia and iron deficiency anaemia at the end of the malaria season in models adjusted for age, sex, village, inflammation and malaria parasitaemia.

**Conclusion:** We found limited statistical evidence linking *IFNG* polymorphisms with a risk of developing iron deficiency and anaemia in Gambian children. More definitive studies are needed to investigate the effects of genetically influenced IFN-γ levels on the risk of iron deficiency and anaemia in children living in malaria-endemic areas.

## Introduction

Malaria and iron deficiency are major public health problems for children living in sub-Saharan Africa. The majority (94%) of the 405,000 global deaths due to malaria in 2018 occurred in sub-Saharan Africa, where up to 24% of the population have malaria parasitaemia at any given time ^1, 2^. In this region, iron deficiency (ID) and anaemia are highly prevalent ^3, 4^, and may lead to impaired brain development ^5, 6^, while iron deficiency anaemia (IDA) is a leading cause of years lived with disability in African children ^7^. Increasing evidence suggests that malaria may be contributing to ID and IDA ^8, 9^. Previous studies reported that the prevalence of ID and IDA increased over the malaria season in Gambian and Kenyan children ^10, 11^, and decreased with the interruption of malaria transmission in the Kenyan highlands ^12^.

Multiple factors may contribute towards the development of ID and IDA following a malaria infection. One such factor is interferon-gamma (IFN-γ), which is induced during acute and persistent malaria infection ^13^. Among other type 1 responses, IFN-γ is involved in regulating erythropoiesis ^14– 16^ and iron-regulatory proteins ^17– 20^. IFN-γ has also been reported to increase the expression of hepcidin ^17^, and divalent metal transporter 1 (DMT1) ^19^, while suppressing ferroportin ^18, 19^, ferritin ^20^, and transferrin receptors ^19, 20^. This regulation of iron proteins may be aimed at starving invading pathogens of iron, a critical nutrient for pathogen growth, but could also play an important role in the pathogenesis of ID and IDA. Indeed, higher IFN-γ levels have been reported in Kenyan children with severe malarial anaemia ^21^.

Single nucleotide polymorphisms (SNPs) in the IFN-γ gene ( *IFNG*) on chromosome 12q14 have been associated with increased production of IFN-γ ^22, 23^, and with susceptibility to severe malaria ^24, 25^. Despite evidence that malaria induces the production of IFN-γ ^13^ and that this cytokine influences iron regulation ^17– 20^, it is not known whether variation in the *IFNG* gene influences the risk of ID and IDA among children in malaria-endemic areas. We investigated SNPs and haplotypes in the *IFNG* gene locus in relation to nutritional iron status and anaemia in a cohort of 780 Gambian children prior to and at the end of a malaria season, using an approach based on informative SNPs and Agena Bioscience MassArray platform typing.

## Methods

### Study area

The study was conducted in ten rural villages in the West Kiang region of The Gambia at the start (July 2001) and end (December 2001/January 2002) of a malaria season, as previously described ^26^. Malaria incidence is highly seasonal in The Gambia, with the majority of cases occurring between September and December. The study participants were from the Mandinka and Fulani ethnic groups. All of the Fulani children were located in a single village and ethnic group was accounted for in all analyses by adjusting for village.

### Study design

We used previously collected data from a cohort of 780 children aged two to six years, recruited at the start of a malaria season as previously described ^26^. All children had a clinical examination and a blood sample collected in the morning between 6 and 11 am for full blood count, malaria film, and biomarkers of iron status and inflammation at the start and end of the malaria season. Children with pyrexia (temperature ≥37.5°C) had appropriate clinical investigations, clinical treatment and a blood sample taken 2 weeks later after recovery from illness. All children received a 3-day course of mebendazole for possible hookworm infection at recruitment.

### Laboratory procedures

Haemoglobin (Medonic CA 530 Haemoglobinometer) and zinc protoporphyrin (ZnPP) levels (Aviv Biomedical Hematoflurometer) were measured within 24 hours of sample collection. Hepcidin (Hepcidin-25 [human] EIA kit; Bachem), ferritin (IMx ferritin assay, Microparticle Enzyme Immunoassay; Abbot Laboratories), soluble transferrin receptor (sTfR, Human sTfR ELISA; R&D Systems), serum iron, unsaturated iron binding capacity (UIBC, Ferrozine-based photometry and colorimetry; Hitachi 911 automated analyzer), and α _1_-antichymotrypsin (ACT, immunoturbidimetry, Cobas Mira Plus Bioanalyzer, Roche) were assayed according to the manufacturers’ instructions from plasma samples stored at -80°C. Transferrin saturation (TSAT) was calculated from plasma iron and UIBC (TSAT = [plasma iron/ (UIBC + plasma iron)] X 100) ^27^. Hepcidin/ferritin and TSAT/hepcidin ratios were also calculated ^28^. Giemsa-stained thick and thin blood films were examined for *Plasmodium falciparum* and other *Plasmodium* species at the start and end of the malaria season.

### SNPs and haplotype construction

Genotypes were determined on whole-genome amplified DNA (primer extension pre-amplification) by the Agena Bioscience MassArray platform (formerly SEQUENOM) using matrix-assisted laser desorption/ionization time-of-flight (MALDI-TOF) mass spectrometry as previously described ^25^. Details of the primer sequences and assays are given in *Extended datafiles 1* and *2* ^29^. The most informative haplotype-tagging SNPs (htSNPs) to type in Gambian subjects were identified by analysing the pattern of linkage disequilibrium (LD) in the *IFNG* gene loci using previously available data from 32 Gambian family trios ^25, 30^. The PHASE program ( http://stephenslab.uchicago.edu/software.html) version 2.1 was used to infer haplotypes from the genotypes of the study population and estimate the frequency of each inferred haplotype ^31^. The entropy maximization method was used to identify htSNPs that described >90% of the observed haplotypic diversity in this gene region ^30^. The HaploXT program ( http://www.sph.umich.edu/csg/abecasis/GOLD/docs/haploxt.html) was used to estimate pairwise LD statistics. Sickle cell (HbS, rs334) and glucose-6-phosphate deficiency (G6PD) deficiency (rs1050828 and rs1050829) were also genotyped using the Agena Bioscience MassArray platform.

### Definition of terms

Inflammation was defined as ACT >0.6 g/L. ID was defined as ferritin <12 µg/L or <30 µg/L in the presence of inflammation or <15 µg/L in children ≥5 years ^32^, anaemia as Hb<11.0 g/dL (or Hb <11.5 g/dL in children ≥5 years) and IDA as ID plus anaemia ^33^.

### Statistical analyses

Statistical analyses were conducted using STATA 15.1 (StataCorp. College Station, Texas, USA). Categorical data were expressed as proportions with corresponding percentages. Pearson chi-squared test was used to compare the prevalence of malaria and iron status (ID, IDA and anaemia) at the start and end of the malaria season. Changes in haemoglobin levels and markers of iron status over the malaria season were assessed using the paired t-test. Biological data that were not normally distributed were log-transformed, and geometric means were calculated from original untransformed values.

Log-transformed markers of iron status and risk of ID, IDA and anaemia were analysed using univariable and multivariable linear and logistic regression models, as appropriate. Multivariable regression models were adjusted for age (grouped by year), sex, village (which also acted as a proxy for ethnic group), malaria parasitaemia and ACT at the start and end of the malaria season. The Bonferroni correction for multiple testing ^34^ was applied when the five SNPs and six haplotypes were considered individually as independent factors. For multivariable analyses, P values are noted as adj. P for non-Bonferroni corrected analyses and as Bonferroni adj. P for multivariable analyses that are Bonferroni corrected, and for univariable models P values are similarly presented as P or Bonferroni P if Bonferroni corrected. All analyses were considered statistically significant at P <0.05.

### Ethics

Individual written informed consent was obtained from children’s parents or guardians and the study was approved by The Gambian Government and the Medical Research Council Ethics Review Committee (874/830).

## Results

### Characteristics of participants

A total of 756 children, including 403 males (53%) and 353 females (47%), were followed up to the end of the malaria season. Most of the children were from the Mandinka ethnic group (n = 681; 90%, compared to Fulani n = 75; 10%). A total of 99/751 (13.2%) children carried sickle cell trait (HbAS) and 136/683 (19.9%) children G6PD deficiency and the effects of these polymorphisms on iron status and anaemia are shown in *Extended datafile* 3 ^29^. At the start of the malaria season we found little difference in iron status in children with HbAS and G6PD deficiency compared to those with wild-type. At the end of the malaria season, children carrying HbAS had lower zinc protoporphyrin levels (97.0 [95% CI 87.0, 108.2]) compared to those with HbAA (115.9 [95% CI 110.6, 121.3]; adj. P = 0.05) and children with G6PD deficiency had lower haemoglobin levels (9.7 g/dl [95% CI 9.4, 10.0]) than those with G6PD wild-type genotype (10.0 g/dl [95% CI 9.9, 10.2]; adj. P = 0.01). The prevalence of ID and IDA increased over the malaria season (from 20.6% to 31.6% and from 11.9% to 21.7%, respectively), as previously reported ^11, 26^. Individual markers of iron status also reflected an increase in ID over the malaria season. We found that hepcidin and hepcidin/ferritin ratio decreased while the TSAT/hepcidin ratio increased across the malaria season in keeping with the need for increased erythropoiesis and increased rates of iron absorption at the end of the malaria season. Table 1 summarises the characteristics of the study population and their iron status at the beginning and end of the malaria season.

**Table 1.** Participant characteristics and iron status at the start and end of the malaria season.

Characteristic	Start	End
Age months, median (IQR)	46.11 (34.83, 59.17)	51.37 (40.47, 64.20)
Sex, Female (%)	353/756 (46.7%)	322/700 (46.0%)
Malaria parasitaemia (%)	89/754 (11.8%)	179/698 (25.6%)
Iron status *		
Iron deficiency ^a^	151/734 (20.6%)	216/ 684 (31.6%)
Iron deficiency anaemia ^b^	86/720 (11.9%)	146/673 (21.7%)
Anaemia ^c^	475/736 (64.5%)	504/691 (72.9%)
Iron biomarkers **		
Hepcidin (ng/ml)	11.1 (9.9, 12.4)	4.2 (3.7, 4.8)
Hepcidin/ferritin	0.44 (0.40, 0.48)	0.20 (0.18, 0.23)
TSAT/hepcidin (%/ng/ml)	0.20 (0.18, 0.23)	1.16 (1.03, 1.31)
Ferritin (µg/L)	25.2 (23.6, 26.8)	20.6 (19.1, 22.2)
ZnPP (µmol/mol Hb)	86.4 (82.9, 90.0)	113.4 (108.7, 118.3)
sTfR (mg/L)	3.45 (3.36, 3.54)	4.16 (4.05, 4.28)
UIBC (µmol/L)	56.8 (55.7, 57.8)	62.7 (61.3, 64.2)
Serum iron (µmol/L)	8.6 (8.3, 9.0)	8.4 (8.2, 8.7)
TSAT (%)	12.9 (12.4, 13.4)	11.6 (11.1, 12.1)
Hb (g/dL)	10.6 (10.5, 10.7)	10.0 (9.8, 10.1)

^*^ Frequency and percentages are shown. ** Geometric means and 95% CIs are shown. ^a^Iron deficiency was defined as ferritin <12µg/l (or ferritin <30µg/l in the presence of inflammation or <15 µg/L in children ≥ 5years) ^32^; ^b^ iron deficiency anaemia as iron deficiency ^a^ and anaemia ^c^; ^c^ anaemia as haemoglobin<11.0 g/dL or haemoglobin <11.5 g/dL in children ≥ 5years. ZnPP, zinc protopophyrin; TSAT, transferrin saturation; sTfR, soluble transferrin receptor; UIBC, unsaturated iron binding capacity; and Hb, haemoglobin.

### *IFNG* single nucleotide polymorphisms and associations

**Figure 1.**
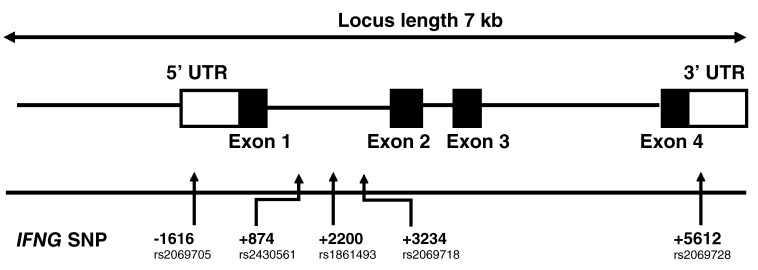
Schematic representation of the *IFNG* gene locus. The *IFNG* single nucleotide polymorphisms (SNP) are designated according to the nucleotide position relative to the transcriptional starting site of *IFNG*.

We identified five *IFNG* haplotype tagging SNPs at positions *IFNG*-1616 (rs2069705), *IFNG*+874 (rs2430561), *IFNG*+2200 (rs1861493), *IFNG*+3234 (rs2069718) and *IFNG*+5612 (rs2069728) relative to the ATG start codon ( Figure 1, *Extended datafile 1* ^29^). All of the *IFNG* SNPs were in Hardy Weinberg equilibrium. The *IFNG*+2200C (rs1861493, n=97) allele was associated with a reduced risk of malaria parasitaemia at the end of the malaria season (adj. OR 0.40 [95% CI 0.21, 0.77]; Bonferroni adj. P=0.03) but not at the start. The other *IFNG* SNPs were not associated with malaria parasitaemia at either time point ( *Extended datafile 4* ^29^). The *IFNG* SNPs were not associated with HbAS or G6PD genotypes following Bonferroni correction in adjusted models. The *IFNG*+874T and *IFNG*+3234C alleles were associated with the Fulani ethnic group (OR 2.22 [95% CI 1.49, 3.31] Bonferroni P = 0.0005 and OR 1.56 [95% CI 1.09, 2.24]; Bonferroni P = 0.045, respectively). The minor allele frequencies by ethnic group are presented in *Extended datafile 1* ^29^.

### Associations with iron and anaemia

We found that the *IFNG* SNPs were not associated with ID, IDA or anaemia at the start of the malaria season in multivariable logistic regression analyses adjusted for age, sex, village, ACT and malaria parasitaemia following Bonferroni adjustment ( *Extended datafile 5* ^29^).

The *IFNG* SNPs were similarly not significantly associated with ID, IDA or anaemia at the end of the malaria season after Bonferroni correction. The *IFNG*+2200C allele (rs1861493) was associated with trends towards increased risk of anaemia (adj. OR 1.86 [95% CI 1.02, 3.38]; adj. P = 0.04), IDA (adj. OR 1.81 [95% CI 1.01, 3.25]; adj. P = 0.05), and ID (adj. OR 1.63 [95% CI 0.95, 2.80]; adj. P = 0.08) in multivariable logistic regression models, but not after Bonferroni correction ( Table 2). Children carrying the *IFNG*+2200C allele had lower haemoglobin levels (9.7 g/dl [95% CI 9.4, 10.1]) compared to those with the *IFNG*+2200 TT genotype (10.0 g/dl [95% CI 9.8, 10.1]; adj. P = 0.006 and Bonferroni adj. P = 0.03), as well as a trend towards ID in other markers of iron status at the end of the malaria season in adjusted linear regression models ( Table 3).

**Table 2.** *IFNG* genotypes and risk of iron deficiency, iron deficiency anaemia and anaemia at the end of the malaria season.

dbSNP number	Genotype	No (%) ^a^	Iron Deficiency ^b^			Iron Deficiency Anaemia ^c^			Anaemia ^d^		
			OR (95% CI) ^f^	Adj P ^f^	Bf adj P ^*^	OR (95% CI) ^¶^	Adj P ^¶^	Bf adj P ^*^	OR (95% CI) ^¶^	Adj P ^¶^	Bf adj P ^*^
rs2069705	IFNG -1616 CC	206 (29.9)	Reference			Reference			Reference		
	IFNG -1616 CT	332 (48.2)	0.82 (0.53, 1.28)	0.40	1	0.62 (0.38, 1.02)	0.06	0.31	0.96 (0.59, 1.53)	0.85	1
	IFNG -1616 TT	151 (21.9)	0.60 (0.34, 1.05)	0.07	0.37	0.53 (0.32, 1.09)	0.09	0.45	0.88 (0.50, 1.55)	0.66	1
rs2430561	IFNG+874 AA	509 (71.4)	Reference			Reference			Reference		
	IFNG+874 AT	182 (25.5)	0.71 (0.44, 1.14)	0.22	0.76	0.75 (0.45, 1.25)	0.27	1	0.93 (0.59, 1.48)	0.76	1
	IFNG+874 TT	22 (3.1)	0.29 (0.09, 0.99)	0.05	0.24	0.37 (0.09, 1.44)	0.15	0.75	0.62 (0.24, 1.63)	0.33	1
rs1861493 ^e^	IFNG +2200 TT	598 (86.0)	Reference			Reference			Reference		
	IFNG +2200 CT/CC	97 (14.0)	1.63 (0.95, 2.80)	0.08	0.38	1.81 (1.01, 3.25)	0.05	0.23	1.86 (1.02, 3.38)	0.04	0.22
rs2069718	IFNG +3234 TT	393 (54.7)	Reference			Reference			Reference		
	IFNG +3234 CT	268 (37.3)	0.87 (0.58, 1.32)	0.52	1	0.89 (0.56, 1.41)	0.62	1	0.95 (0.62, 1.45)	0.82	1
	IFNG +3234 CC	58 (8.1)	0.79 (0.39, 1.60)	0.51	1	0.73 (0.33, 1.63)	0.44	1	0.67 (0.34, 1.32)	0.25	1
rs2069728	IFNG +5612 CC	326 (47.0)	Reference			Reference			Reference		
	IFNG +5612 CT	302 (43.6)	0.85 (0.57, 1.28)	0.44	1	0.76 (0.48, 1.19)	0.22	1	0.94 (0.62, 1.42)	0.76	1
	IFNG +5612 TT	65 (9.4)	0.97 (0.48, 1.96)	0.93	1	0.47 (0.20, 1.12)	0.09	0.45	0.94 (0.45, 1.95)	0.87	1

^a^ Values are number (%) in each group; ^b^ Ferritin <12µg/l (or ferritin<30µg/l in the presence of inflammation or <15 µg/L in children ≥5 years) ^32^; ^c^ Iron deficiency ^b^ and anaemia ^d^; ^d ^Haemoglobin<11.0g/dL (or haemoglobin<11.5g/dL in children ≥5years); ^e ^The mutant alleles for *IFNG*+2200 were combined because of the low frequency of *IFNG*+2200CC (n=4) in the study population; ^¶^ Odds ratios (OR) and P values were derived by multivariable logistic regression adjusted for age, sex, village (a proxy for location and ethnic group), α _1_-antichymotrypsin level and the presence of malaria parasites on blood film; ^*^P values additionally adjusted for Bonferroni correction for multiple testing.

**Table 3.** *IFNG*+2200 SNPs and markers of iron status at the end of the malaria season.

Iron Marker	n	*IFNG*+2200 TT ^a, b^	n	*IFNG*+2200 CT/CC ^a, b^	Adj P ^¶^	Bf adj P *
Hepcidin (ng/ml)	500	4.4 (3.8, 5.1)	84	3.2 (2.2, 4.6)	0.09	0.44
Hepcidin/ferritin	500	0.20 (0.18, 0.23)	84	0.18 (0.14, 0.25)	0.19	0.94
TSAT/hepcidin (%/ng/ml)	490	2.75 (2.38, 3.17)	81	3.33 (2.38, 4.67)	0.87	1
Ferritin (µg/L)	545	21.4 (19.7, 23.2)	91	16.9 (13.6, 21.1)	0.04	0.21
ZnPP (µmol/mol Hb)	550	112.4 (107.2, 117.8)	91	121.2 (107.0, 137.3)	0.06	0.32
sTfR (mg/L)	508	4.2 (4.0, 4.3)	86	4.2 (3.9, 4.6)	0.38	1
UIBC (µmol/L)	530	61.6 (60.1, 63.2)	87	67.9 (62.6, 73.8)	0.04	0.19
Serum iron (µmol/L)	532	8.6 (8.3, 8.9)	88	7.9 (7.1, 8.7)	0.19	0.95
TSAT (%)	528	12.0 (11.4, 12.5)	87	10.1 (8.8, 11.6)	0.04	0.19
Hb (g/dL)	546	10.0 (9.8, 10.1)	92	9.7 (9.4, 10.1)	0.006	0.03

^a^ Values are geometric means (95% confidence interval); ^b^ The wild-type alleles represent children with *IFNG*+2200TT genotype (n=598). The mutant alleles represent children with the *IFNG*+2200TC (n=93) and *IFNG*+2200CC (n=4) genotypes combined; ^¶^ Significance values were derived by multivariable linear regression with each log-transformed iron marker as a dependent variable and adjusted for age, sex, village (a proxy for location and ethnic group), α _1_-antichymotrypsin level and the presence of malaria parasites on blood film. * P values adjusted for Bonferroni correction for the five tested SNPs. n, number of children; Hb, haemoglobin; ZnPP, zinc protopophyrin; sTfR, soluble transferrin receptor; UIBC, unsaturated iron binding capacity; and TSAT, transferrin saturation.

### *IFNG* haplotypes

Haplotype analysis identified ten haplotypes (four with less than 1% population frequency) resolved by SNPs at nucleotide positions -1616, +874, +2200, +3234 and +5612 in the *IFNG* gene locus ( Figure 1). Six haplotypes accounted for most of the variation. The wild-type haplotype (haplotype 1, *IFNG*-CATTC) was present at a frequency of 35% in the Gambian children, while haplotype 6 ( *IFNG*-CACTC), uniquely identified by the *IFNG*+2200 SNP, was present at a frequency of 7%. Using haplotype 1 as the reference, haplotype 6 was associated with a trend towards increased risk of ID (adj. OR 1.58 [95% CI 0.93, 2.69]), IDA (adj. OR 1.71 [95% CI 0.94, 3.10]) and anaemia (adj. OR 1.67 [95% CI 0.93, 3.01]) at the end of the malaria season ( Table 4). Haplotype 6 was also associated with reduced haemoglobin concentrations (adj. β -0.48 [95% CI -0.79, -0.18]; P = 0.002) and TSAT (adj. β -0.15 [95% CI -0.27, -0.03]; P = 0.02), and higher ZnPP levels (adj. β 0.06 [95% CI 0.01, 0.12]; P = 0.02) and a trend towards reduced ferritin levels compared to the wild-type haplotype ( Table 5).

**Table 4.** *IFNG* haplotypes and risk of iron deficiency, iron deficiency anaemia and anaemia at the end of the malaria season.

Hap ID	Haplotype ^a^	Freq (No)	Iron Deficiency ^b^		Iron Deficiency Anaemia ^c^		Anaemia ^d^	
			OR (95% CI) ^¶^	Adj P ^¶^	OR (95% CI) ^¶^	Adj P ^¶^	OR (95% CI) ^¶^	Adj P ^¶^
Hap1	CATTC	0.35 (536)	Reference		Reference		Reference	
Hap2	**T**ATT ** T**	0.19 (283)	0.82 (0.55, 1.21)	0.31	0.69 (0.44, 1.07)	0.10	1.08 (0.72, 1.60)	0.72
Hap3	**TT**T **C**C	0.16 (240)	0.70 (0.46, 1.06)	0.09	0.68 (0.43, 1.08)	0.10	0.83 (0.56, 1.24)	0.37
Hap4	CATT ** T**	0.11 (171)	1.14 (0.73, 1.78)	0.73	0.91 (0.53, 1.56)	0.73	0.78 (0.50, 1.23)	0.28
Hap5	**T**AT **C**C	0.11 (166)	1.19 (0.77, 1.85)	0.58	0.91 (0.53, 1.55)	0.73	1.03 (0.65, 1.66)	0.89
Hap6	CA **C**TC	0.07 (100)	1.58 (0.93, 2.69)	0.09	1.71 (0.94, 3.10)	0.09	1.67 (0.93, 3.01)	0.09

^a^ The haplotype configuration is as follows: *IFNG*-1616, *IFNG*+874, *IFNG*+2200, *IFNG*+3234, *IFNG*+5612. Minor alleles are indicated by bold type; ^b^ ferritin <12µg/l (or ferritin<30µg/l in the presence of inflammation or <15 µg/L in children ≥5 years) ^32^; ^c^ iron deficiency and anaemia; ^d^ haemoglobin<11.0g/dL (or haemoglobin<11.5g/dL in children ≥5years); ^¶^ odds ratios (OR) and P values were derived by multivariable logistic regression adjusted for age, sex, village, α1-antichymotrypsin level and malaria parasitaemia. Hap, haplotype; Freq, frequency of the haplotypes in the study population; no, number of haplotype alleles in the study population.

**Table 5.** *IFNG* haplotypes and markers of iron status at the end of the malaria season.

Hap	Hepcidin (ng/ml)	AdjP ^¶^	Ferritin (µg/L)	Adj P ^¶^	ZnPP (µmol/mol Hb)	Adj P ^¶^	TSAT (%)	Adj P ^¶^	sTfR (mg/L)	Adj P ^¶^	Hb (g/dl)	Adj P ^¶^
CATTC	4.3 (3.7, 5.0)	Ref	19.4 (17.8, 21.1)	Ref	115.3 (109.6, 121.3)	Ref	11.5 (11.0, 12.1)	Ref	4.2 (4.0, 4.3)	Ref	10.0 (9.8, 10.1)	Ref
**T**ATT **T**	4.5 (3.5, 5.6)	0.69	22.3 (19.7, 25.3)	0.06	113.0 (105.0, 121.6)	0.41	11.7 (11.0, 12.6)	0.93	4.2 (4.0, 4.4)	0.76	9.9 (9.7, 10.1)	0.22
**TT**T **C**C	4.3 (3.3, 5.5)	0.95	24.4 (21.3, 27.9)	0.05	115.1 (107.3, 123.5)	0.54	11.3 (10.4, 12.4)	0.58	4.1 (3.9, 4.4)	0.50	10.0 (9.7, 10.2)	0.85
CATT **T**	5.2 (4.0, 6.7)	0.28	20.9 (17.8, 20.5)	0.54	103.8 (95.9, 112.4)	0.26	12.9 (11.9, 14.0)	0.09	4.0 (3.8, 4.3)	0.30	10.2 (9.9, 10.4)	0.31
**T**AT **C**C	3.5 (2.6, 4.7)	0.16	18.8 (16.0, 22.1)	0.64	109.3 (99.5, 120.1)	0.87	11.5 (10.5, 12.6)	0.92	4.2 (4.0, 4.5)	0.80	10.0 (9.8, 10.3)	0.74
CA **C**TC	3.2 (2.3, 4.6)	0.11	16.6 (13.4, 20.5)	0.08	123.5 (109.1, 139.8)	0.02	9.8 (8.5, 11.3)	0.02	4.2 (3.9, 4.6)	0.28	9.7 (9.3, 10.0)	0.002

Values are geometric means (95% confidence interval). ^¶ ^Significance (P) values were derived by multivariable linear regression with each log-transformed iron marker as a dependent variable and adjusted for age, sex, village (a proxy for location and ethnic group), α1-antichymotrypsin level and the presence of malaria parasites on blood film. Hap, haplotype; Hb, haemoglobin; ZnPP, zinc protopophyrin; sTfR, soluble transferrin receptor; and TSAT, transferrin saturation.

## Discussion

In this study we observed an increase in the prevalence of ID, IDA and anaemia across the malaria season in Gambian children. Dietary iron insufficiency may be an important cause since the malaria season also coincides with the ‘hungry season’ in The Gambia when there is a scarcity of staple foods. We hypothesized that IFN-γ, a pro-inflammatory cytokine induced during malaria infection ^13^, might play a role in influencing the risk of ID and anaemia in children exposed to malaria. In addition to directly reducing erythrocyte half-life ^15^, evidence suggests that IFN-γ induces hepcidin and inhibits ferroportin, hence reducing iron absorption and promoting sequestration of iron in macrophages ^17, 19^. Consequently, high levels of IFN-γ induced during malaria infections may concomitantly lead to ID and anaemia.

We found that the *IFNG*+2200C (rs1861493) allele, located at intron 3 of the *IFNG* gene, was associated with reduced haemoglobin levels and a trend towards ID, IDA and anaemia at the end of a malaria season in multivariable analyses adjusting for potential confounders. We then constructed haplotypes to increase the probability of capturing functional mutations which might reside within a given haplotype. Haplotype 6 (uniquely identified by the *IFNG*+2200C allele), was associated with reduced haemoglobin levels and TSAT and increased ZnPP levels in keeping with iron deficiency compared to the wild-type haplotype. Haplotype 6 was similarly associated with trends towards increased risk of ID, IDA and anaemia at the end of the malaria season.

So how might the *IFNG*+2200C genotype and a haplotype uniquely defined by this genotype potentially lead to reduced haemoglobin levels and ID at the end of the malaria season? A possible explanation may be through increasing *IFNG* gene expression and IFN-γ levels. The *IFNG*+2200C allele was associated with increased IFN-γ levels in Kawasaki disease patients ^23^, although another study in patients with ankylosing spondylitis found no difference in IFN-γ levels by *IFNG*+2200 genotype ^35^. Elevated IFN-γ levels promote dyserythropoiesis, anaemia and iron dysregulation. IFN-γ inhibits proliferation of erythroid progenitor cells by disrupting lineage differentiation, blocking renal production of erythropoietin, inhibiting renal iron reabsorption, and reducing red blood cell half-life ^14– 16, 36^. As a type 1 immune response, IFN-γ also induces defensive transcriptional programs within enterocytes resulting in reduced dietary iron absorption ^37^. Additionally, IFN-γ promotes iron sequestration in macrophages either directly or through its influence on hepcidin, ferroportin, and DMT1 ^17– 20^. These responses reduce circulating transferrin-bound iron, which is required by *Plasmodium* parasites for metabolism and proliferation ^38^. In keeping with this, we observed decreased ferritin levels and TSAT and increased ZnPP levels in children carrying the *IFNG*+2200C haplotype. Lower haemoglobin levels may also translate into reduced amino acid availability for *Plasmodium* parasites ^39^ and hence protection against blood-stage parasitaemia. Indeed, we observed that the *IFNG*+2200C SNP was associated with protection against malaria parasitaemia at the end of the malaria season (adj. OR 0.40; Bonferroni adj. P=0.03).

The influence of the *IFNG*+2200C allele on haemoglobin and iron status was only observed at the end of the malaria season. We hypothesized that the effects of this SNP may be most marked when expression of *IFNG* is upregulated, such as during malaria infections ^13, 24^. This also highlights the influence of gene-environment interactions in promoting disease, in this case ID, IDA and anaemia. It is unlikely that the decreased haemoglobin levels observed in individuals carrying the *IFNG*+2200C allele was due to increased malaria since these children had reduced prevalence of malaria parasitaemia at the end of the malaria season. It is possible that higher IFN-γ levels, putatively produced by *IFNG*+2200C carriers, may induce a protective proinflammatory response against malaria ^13, 40^, but at the expense of iron homeostasis. The *IFNG*+874TT (rs2430561) genotype, located at the first intron coinciding with the NFkB binding region, has also been associated with higher production of IFN-γ ^22, 35, 41^. However, studies have found no association between *IFNG*+874TT and malaria ^25^ or aplastic anaemia ^42^, and in our study we observed a trend towards a decreased risk of ID in these individuals. Further investigations are required on a cellular level to explore putative functional effects of *IFNG* genotypes on IFN-γ levels and iron status.

To our knowledge, this is the first study examining the role of *IFNG* gene polymorphisms in relation to iron status. We found that the *IFNG*+2200C (rs1861493) allele, and a haplotype defined by this allele, were associated with reduced haemoglobin levels and a trend towards ID at the end of the malaria season, a finding that may be due to increased IFN-γ levels ^23^. However, our study had a number of important limitations and our findings should be viewed with considerable caution. The study was conducted in a single site and had relatively small numbers (n = 756). Additionally, many of our findings were of marginal significance with wide confidence intervals and lost statistical significance after correction for multiple testing with Bonferroni adjustment. It is also unclear if our findings have clinical relevance at an individual level. The *IFNG*+2200C SNP may also be in linkage disequilibrium (LD) with another genetic variant within the haplotype that might influence IFN-γ levels and / or measures of iron status in Gambian populations. Finally, we did not measure IFN-γ levels to determine if they differed between genotypes at the end of the malaria season. Thus, our findings need to be examined in larger population-based studies, in other malaria-exposed populations, and functional assays are needed to identify whether genetic variation in the *IFNG* gene influences iron status. However, our study supports the hypothesis that preventing and treating malaria infection may improve haemoglobin levels and iron status in African children ^8^.

## Data availability

### Underlying data

Havard Dataverse: Replication Data for: Interferon-gamma polymorphisms and risk of iron deficiency and anaemia in Gambian children, https://doi.org/10.7910/DVN/2NKJID ^43^. This project contains the following underlying data:

- IFNG_final_data_v2 (dataset containing demographic information and results of laboratory assays for participants included in the study).
- IFNG_analysis_KM (contains the codes used for data analysis).
- KMokaya_IFNG_Codebook (contains variable description and labels).
- Data are available under the terms of the Creative Commons Attribution 4.0 International license (CC-BY 4.0).

### Extended Data

Figshare: *IFNG* polymorphisms in Gambian children and risk of iron deficiency and anaemia, https://doi.org/10.6084/m9.figshare.11807277.v6 ^29^.

This project contains the following extended data:

Extended datafile 1. Assayed *IFNG* single nucleotide polymorphisms

Extended datafile 2. Agena Biosciences (formerly SEQUENOM) MassARRAY ^®^ primer-extension definitions data for the five *IFNG* polymorphisms

Extended datafile 3. Markers of iron status at the end of the malaria season in children with sickle cell trait and glucose-6-phosphate dehydrogenase deficiency

Extended datafile 4. *IFNG* single nucleotide polymorphisms and risk of malaria parasitemia at the start and end of the malaria season

Extended datafile 5. *IFNG* genotypes and risk of iron deficiency, iron deficiency anaemia and anaemia at the start of the malaria season

Data are available under the terms of the Creative Commons Attribution 4.0 International license (CC-BY 4.0).
