# Supplementary material for: Interferon-gamma polymorphisms and risk of iron deficiency and anaemia in Gambian children
Source: Wellcome Open Res. 2020 Jun 2;5:40. Originally published 2020 Mar 2. [Version 2] doi: 10.12688/wellcomeopenres.15750.2 (PMC7202087; doi:10.12688/wellcomeopenres.15750.2)
Supplement: Peer review history

# Peer review: Reviewer response for version 1

**DOI:** 10.21956/wellcomeopenres.17270.r38445

**Published:** 2020-05-05

**Authors:** Laura Silvestri

**Affiliations:** 1 Regulation of Iron Metabolism Unit, Division of Genetics and Cell Biology, San Raffaele Scientific Institute, Milan, Italy

In this paper Abuga et al investigate the role of IFNG SNPs in influencing iron deficiency (ID) and iron deficiency anemia (IDA) in Gambian children over a malaria season. They identify a SNP, IFNG+2200C, located in intron 3, associated with a reduced risk of malaria parasitemia, reduced Hb, and a trend towards ID and IDA. The authors conclude that although more studies are needed to assess the role of IFNG SNPs in ID and IDA, this represents the first study that investigates the association of IFNG genetic variants with iron status.

 The study is well conducted and of interest. I have only minor comments:

1. Serum hepcidin levels are influenced by circadian rhythm and serum/iron stores. Please indicate at what time of the day blood was drawn. To “normalize” serum hepcidin to body iron concentration, the hepcidin/ferritin or hepcidin/TSAT ratio should be shown in Table 1 and Table 3 (as in Donker et al., Pediatric Blood and Cancer 2019 ^1^).
2. In the M&M section, the authors claim that some children are carriers of the HbAS, and G6PD deficiency. Is it possible to present the hematological and iron data (in Table 1 and Table 3) related to these children?

Is the work clearly and accurately presented and does it cite the current literature?

Yes

If applicable, is the statistical analysis and its interpretation appropriate?

I cannot comment. A qualified statistician is required.

Are all the source data underlying the results available to ensure full reproducibility?

Yes

Is the study design appropriate and is the work technically sound?

Yes

Are the conclusions drawn adequately supported by the results?

Yes

Are sufficient details of methods and analysis provided to allow replication by others?

Yes

Reviewer Expertise:

Laura Silvestri has long-term expertise in the characterization of molecular mechanisms responsible for the pathogenesis of iron/hepcidin-related disorders as Hereditary Hemochromatosis, beta-thalassemia, and iron refractory iron deficiency anemia (IRIDA).

I confirm that I have read this submission and believe that I have an appropriate level of expertise to confirm that it is of an acceptable scientific standard.

## References

[1] : Standardized serum hepcidin values in Dutch children: Set point relative to body iron changes during childhood. *Pediatr Blood Cancer*.67(3) : 10.1002/pbc.28038 e28038 10.1002/pbc.28038 31724793

# Response

**Published:** 2020-05-27

**Authors:** Sarah Atkinson

**Affiliations:** KEMRI-Wellcome Research Programme, Kenya

Thank you for your helpful comments. 

**a) Serum hepcidin levels are influenced by circadian rhythm and serum/iron stores. Please indicate at what time of the day blood was drawn.**

*Response: Thank you, Blood was drawn from the participants in the morning hours (between 6 and 11AM). We have updated the study design section to indicate the time of sample collection.*

*Methods, under Study design:* “ *All children had a clinical examination and a blood sample collected in the morning between 6 and 11 am for full blood count, malaria film, and biomarkers of iron status and inflammation at the start and end of the malaria season.”*

**b) To “normalize” serum hepcidin to body iron concentration, the hepcidin/ferritin or hepcidin/TSAT ratio should be shown in Table 1 and Table 3 (as in Donker et al., Pediatric Blood and Cancer 2019).**

*Response: Thank you for the suggestion. We have added hepcidin/ferritin and transferrin saturation (TSAT)/hepcidin ratios to tables 1 and 3. We have also made the following changes:*

*Methods section under laboratory methods: Hepcidin/ferritin and TSAT/hepcidin ratios were also calculated*. ^1^

*Results section under Characteristics of participants: We found that hepcidin and hepcidin/ferritin ratio decreased while the TSAT/hepcidin ratio increased across the malaria season in keeping with the need for increased erythropoiesis and increased rates of iron absorption at the end of the malaria season.*

**2. In the M&M section, the authors claim that some children are carriers of the HbAS, and G6PD deficiency. Is it possible to present the haematological and iron data (in Table 1 and Table 3) related to these children?**

*Response: Thank you for this suggestion. We have now added haemoglobin and iron biomarker data for children with HbAS and G6PD deficiency as a Supplementary file (Extended datafile 3) that can be accessed on Figshare (* *https://doi.org/10.6084/m9.figshare.11807277* *.v6* *). We have also made the following changes to the Results section:*

*Results under Characteristics of participants: “A total of 99/751 (13.2%) children carried sickle cell trait (HbAS) and 136/683 (19.9%) children G6PD deficiency and the effects of these polymorphisms on iron status and anaemia are shown in Extended datafile 3.* ^*2*^ *At the start of the malaria season we found little difference in iron status in children with HbAS and G6PD deficiency compared to those with wild-type. At the end of the malaria season, children carrying HbAS had lower zinc protoporphyrin levels (97.0 [95% CI 87.0, 108.2]) compared to those with HbAA (115.9 [95% CI 110.6, 121.3]; adj. P = 0.05) and children with G6PD deficiency had lower haemoglobin levels (9.7 g/dl [95% CI 9.4, 10.0]) than those with G6PD wild-type genotype (10.0 g/dl [95% CI 9.9, 10.2]; adj. P = 0.01).”*

**References**

 1.         Donker AE, Galesloot TE, Laarakkers CM, Klaver SM, Bakkeren DL, Swinkels DW. Standardized serum hepcidin values in Dutch children: Set point relative to body iron changes during childhood. *Pediatr Blood Cancer* 2020; **67**(3): e28038.

 2.         Abuga KM, Robert KA, Muriuki JM, et al. IFNG polymorphisms in Gambian children and risk of iron deficiency and anaemia. 2020. https://doi.org/10.6084/m9.figshare.11807277.

# Peer review: Reviewer response for version 1

**DOI:** 10.21956/wellcomeopenres.17270.r38239

**Published:** 2020-04-23

**Authors:** John Michael Ong’echa

**Affiliations:** 1 Center for Global Health Research, Kenya Medical Research Institute (KEMRI), Kisumu, Kenya

**Abstract:**

 It would be good if the authors provided the age range of children enrolled in the study (2 – 6 years).  This could be added in the Methods section at the end of the sentence ‘….780 Gambian children.’

 The last sentence at the end of the Results section mentions a number of covariates that were adjusted for in the models. One such covariate is ‘malaria parasitaemia’.

 Was malaria parasitaemia adjusted for only at the beginning and at the end of the malaria season or did it capture all the malaria cases during the study period? Does ‘malaria parasitaemia’ mean malaria episodes during the study period?  If it only captures the two time points, what would the effect of ‘additional episodes’ during the study period have on the results? In malaria endemic areas, repeated infections are more likely to result in anaemia than just the patent infection. 

 The authors need to clarify on this and if there were no malaria cases in-between the two time-points they need to indicate so.    

Is the work clearly and accurately presented and does it cite the current literature?

Yes

If applicable, is the statistical analysis and its interpretation appropriate?

Yes

Are all the source data underlying the results available to ensure full reproducibility?

Yes

Is the study design appropriate and is the work technically sound?

Yes

Are the conclusions drawn adequately supported by the results?

Yes

Are sufficient details of methods and analysis provided to allow replication by others?

Yes

Reviewer Expertise:

NA

I confirm that I have read this submission and believe that I have an appropriate level of expertise to confirm that it is of an acceptable scientific standard.

# Response

**Published:** 2020-05-27

**Authors:** Sarah Atkinson

**Affiliations:** KEMRI-Wellcome Research Programme, Kenya

We thank the reviewer for his helpful comments, which we have addressed point-by-point below:

**Reviewer #1**

1. **Abstract: It would be good if the authors provided the age range of children enrolled in the study (2 – 6 years).This could be added in the Methods section at the end of the sentence ‘….780 Gambian children.’**

*Response: Thank you for the suggestion. We have added the age range (2-6 years) in the methods section of the abstract.*

*Abstract, Methods: “We used previously available data from Gambian family trios to determine informative SNPs and then used the Agena Bioscience MassArray platform to type five SNPs from the IFNG gene in a cohort of 780 Gambian children aged 2-6 years”.*

**2. The last sentence at the end of the Results section mentions a number of covariates that were adjusted for in the models. One such covariate is ‘malaria parasitaemia’.**

**a) Was malaria parasitaemia adjusted for only at the beginning and at the end of the malaria season or did it capture all the malaria cases during the study period?**

*Response: In the current analysis, we adjusted for malaria parasitaemia detected on blood film at the beginning and at the end of the malaria season, at the same timepoints when iron biomarkers were measured since malaria parasitaemia influences markers of iron status. We did not monitor for malaria cases during the malaria season.*

*Methods, under Statistical analyses: “Multivariable regression models were adjusted for age (grouped by year), sex, village (which also acted as a proxy for ethnic group), malaria parasitaemia and ACT at the start and end of the malaria season.”*

**b)  Does ‘malaria parasitaemia’ mean malaria episodes during the study period?**

*Response: Malaria parasitaemia referred to the identification of Plasmodium parasites on the blood film taken from the study participants at either of the two cross-sectional timepoints, i.e. at the beginning and / or end of the malaria season. It does not refer to malaria episodes during the study period.*

**c) If it only captures the two time points, what would the effect of ‘additional episodes’ during the study period have on the results? In malaria endemic areas, repeated infections are more likely to result in anaemia than just the patent infection. The authors need to clarify on this and if there were no malaria cases in-between the two time-points they need to indicate so.**

*Response: We agree that repeated malaria episodes would increase the risk of anaemia. Based on published data we estimated that the majority of the children would be likely to have had one or more episodes of malaria over the malaria season during the study period.* *^1^* *Studies indicate that malaria increases IFN-* *g* *levels* ^2^ *and that IFN-* *g* *regulates erythropoiesis and iron-regulatory proteins including hepcidin.* ^3-6^ *We therefore hypothesized that IFNG SNPs, which might alter IFNG expression, would influence iron status at the end of the malaria season. Thus, we would expect that the effect of ‘additional episodes’ of malaria would be to increase malaria-induced IFN-* *g* *levels and IFN-* *g* *-induced iron deficiency and anaemia. To clarify we have updated the Methods section as follows:*

*Methods, under Laboratory methods: “Giemsa-stained thick and thin blood films were examined for Plasmodium falciparum and other Plasmodium species at the start and end of the malaria season.”*

# Response

**Published:** 2020-05-27

**Authors:** Sarah Atkinson

**Affiliations:** KEMRI-Wellcome Research Programme, Kenya

We thank the reviewer for his helpful comments, which we have addressed point-by-point below:

  

1. **Abstract: It would be good if the authors provided the age range of children enrolled in the study (2 – 6 years).This could be added in the Methods section at the end of the sentence ‘….780 Gambian children.’**

 

*Response: Thank you for the suggestion. We have added the age range (2-6 years) in the methods section of the abstract.*

*Abstract, Methods: “We used previously available data from Gambian family trios to determine informative SNPs and then used the Agena Bioscience MassArray platform to type five SNPs from the IFNG gene in a cohort of 780 Gambian children aged 2-6 years”.*

**2. The last sentence at the end of the Results section mentions a number of covariates that were adjusted for in the models. One such covariate is ‘malaria parasitaemia’.**

**a) Was malaria parasitaemia adjusted for only at the beginning and at the end of the malaria season or did it capture all the malaria cases during the study period?**

*Response: In the current analysis, we adjusted for malaria parasitaemia detected on blood film at the beginning and at the end of the malaria season, at the same timepoints when iron biomarkers were measured since malaria parasitaemia influences markers of iron status. We did not monitor for malaria cases during the malaria season.*

*Methods, under Statistical analyses: “Multivariable regression models were adjusted for age (grouped by year), sex, village (which also acted as a proxy for ethnic group), malaria parasitaemia and ACT at the start and end of the malaria season.”*

**b) Does ‘malaria parasitaemia’ mean malaria episodes during the study period?**

*Response: Malaria parasitaemia referred to the identification of Plasmodium parasites on the blood film taken from the study participants at either of the two cross-sectional timepoints, i.e. at the beginning and / or end of the malaria season. It does not refer to malaria episodes during the study period.*

**c) If it only captures the two time points, what would the effect of ‘additional episodes’ during the study period have on the results? In malaria endemic areas, repeated infections are more likely to result in anaemia than just the patent infection. The authors need to clarify on this and if there were no malaria cases in-between the two time-points they need to indicate so.**

*Response: We agree that repeated malaria episodes would increase the risk of anaemia. Based on published data we estimated that the majority of the children would be likely to have had one or more episodes of malaria over the malaria season during the study period.* *^1^* *Studies indicate that malaria increases IFN-* *g* *levels* ^2^ *and that IFN-* *g* *regulates erythropoiesis and iron-regulatory proteins including hepcidin.* ^3-6^ *We therefore hypothesized that IFNG SNPs, which might alter IFNG expression, would influence iron status at the end of the malaria season. Thus, we would expect that the effect of ‘additional episodes’ of malaria would be to increase malaria-induced IFN-* *g* *levels and IFN-* *g* *-induced iron deficiency and anaemia. To clarify we have updated the Methods section as follows:*

*Methods, under Laboratory methods: “Giemsa-stained thick and thin blood films were examined for Plasmodium falciparum and other Plasmodium species at the start and end of the malaria season.”*

**References**

 1.         Ceesay SJ, Casals-Pascual C, Erskine J, et al. Changes in malaria indices between 1999 and 2007 in The Gambia: a retrospective analysis. *Lancet (London, England)* 2008; **372**(9649): 1545-54.

 2.         McCall MB, Sauerwein RW. Interferon-gamma--central mediator of protective immune responses against the pre-erythrocytic and blood stage of malaria. *Journal of leukocyte biology* 2010; **88**(6): 1131-43.

 3.         Frazier MD, Mamo LB, Ghio AJ, Turi JL. Hepcidin expression in human airway epithelial cells is regulated by interferon-γ. *Respiratory research* 2011; **12**(1): 100.

 4.         Nairz M, Fritsche G, Brunner P, Talasz H, Hantke K, Weiss G. Interferon-gamma limits the availability of iron for intramacrophage Salmonella typhimurium. *European journal of immunology* 2008; **38**(7): 1923-36.

 5.         Ludwiczek S, Aigner E, Theurl I, Weiss G. Cytokine-mediated regulation of iron transport in human monocytic cells. *Blood* 2003; **101**(10): 4148-54.

 6.         Byrd TF, Horwitz MA. Regulation of transferrin receptor expression and ferritin content in human mononuclear phagocytes. Coordinate upregulation by iron transferrin and downregulation by interferon gamma. *The Journal of clinical investigation* 1993; **91**(3): 969-76.
